# Case Report: Surgical excision of urethral transitional cell carcinoma by total urethrectomy combined with partial cystectomy and vesicovaginal urinary diversion in two female dogs

**DOI:** 10.3389/fvets.2026.1766294

**Published:** 2026-05-04

**Authors:** Wookhun Chung, Kihoon Kim, Jaewoong Han, Byung-Joon Seung, Young-Hyun Goo, Sun-Hee Do

**Affiliations:** 1 Nowon N Animal Medical Center, Seoul, Republic of Korea; 2 Department of Veterinary Surgery, College of Veterinary Medicine, Kangwon National University, Chuncheon, Republic of Korea; 3 Department of Pathology, College of Medicine, Kangwon National University, Chuncheon, Republic of Korea; 4 Department of Veterinary Clinical Pathology, College of Veterinary Medicine, Konkuk University, Seoul, Republic of Korea

**Keywords:** canine, partial cystectomy, total urethrectomy, transitional cell carcinoma, vesicovaginal diversion

## Abstract

Transitional cell carcinoma (TCC), or urothelial carcinoma, is the most common malignant tumor of the canine urinary bladder or urethra, often causing urinary obstruction. While chemotherapy is generally considered as primary management, surgical treatments, including total or partial cystectomy, have occasionally been reported. This report describes two cases of canine urethral TCC managed with total urethrectomy combined with partial cystectomy and vesicovaginal urinary diversion. In both cases, the entire urethral segment and distal urinary bladder were removed while preserving the ureteral orifices. Ventral cystostomy was performed to expose the masses, and special care was taken to maintain vascular supply to the bladder. Postoperative recovery was uneventful. Although chemotherapy was recommended, it was performed in only one case. Urinary flow from the urinary bladder to vagina was successfully diverted without ureteral reimplantation. No recurrence was observed for approximately one year postoperatively in Case 1 and one and a half years in Case 2, respectively. These findings suggest that total urethrectomy combined with partial cystectomy and vesicovaginal urinary diversion is a feasible and effective surgical approach for localized urethral TCC in female dogs.

## 1 Introduction

Bladder neoplasms represent approximately 1–3% of all canine malignancies (1, 2). Most tumors arising in the bladder are of epithelial origin, with transitional cell carcinomas (TCC), also known as urothelial carcinomas, being the most common malignant neoplasm identified in the urinary bladder or urethra in dogs (1, 3, 4). These malignant tumors are primarily located in the trigone of the bladder or urethra and are highly aggressive, often resulting in urine outflow obstruction (4–6). Due to the specific location of the tumors, as well as the field cancerization effect in which multiple independent neoplastic foci may be present throughout the bladder, medical management such as chemotherapy has been the mainstay for treatment of TCC (1, 7). However, total cystectomy with or without proximal urethrectomy for surgical management of bladder (4, 8–10) or urethral (4) tumor has also been occasionally reported in the veterinary medicine. Furthermore, partial cystectomy has been reported, which resulted in bladder necrosis in limited cases (11, 12). To the best of the authors’ knowledge, this is the first report describing total urethrectomy and partial cystectomy followed by vesicovaginal urinary diversion for treatment of urethral TCC without bladder necrosis in veterinary medicine.

## 2 Case description

### 2.1 Case 1

**Figure 1:**
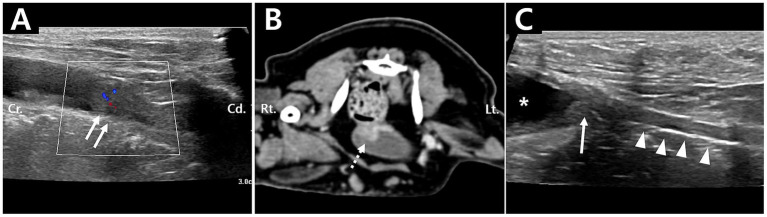
Preoperative (**A**, **B**, Case 1) and postoperative (**C**, Case 2) imaging findings of transitional cell carcinoma. **(A)** Ultrasonographic image showing a mass (white arrow) with mild peripheral vascular flow at the proximal urethra. **(B)** Post-contrast image of a mass (dashed arrow) with mild contrast enhancement at the bladder trigone. **(C)** Postoperative ultrasonographic image obtained 1 year after surgery, demonstrating the bladder (asterisk), vagina (arrowhead), and anastomosis site (arrow).

An 11-year-old, spayed female, weighing 2.5 kg, Maltese was referred to Nowon N Animal Medical Center for suspected urinary bladder tumor. On ultrasonographic examination, an oval-shaped heterogeneously hypoechoic mass (10 × 9.3 × 5.9 mm) was identified from the trigone of the bladder to the proximal urethra, and mild peripheral vascular flow was identified (Figure 1A). On computed tomographic examination, a contrast-enhancing elliptical mass was identified in the trigone of the bladder (Figure 1B), and additionally, a mass with mild contrast enhancement was observed within the proximal urethra. Furthermore, partial loss of normal mural stratification was identified, suggesting invasion beyond the mucosal layer. Although the lesion was adjacent to the left ureteral orifice, no luminal obstruction was observed. Both ureters were clearly identifiable, and there was no evidence of hydroureter or hydronephrosis, indicating preserved ureteral patency at the time of imaging. No definite invasion into adjacent pelvic structures was detected. Additionally, the right sublumbar lymph node was mildly enlarged compared to the left sublumbar lymph node.

**Figure 2:**
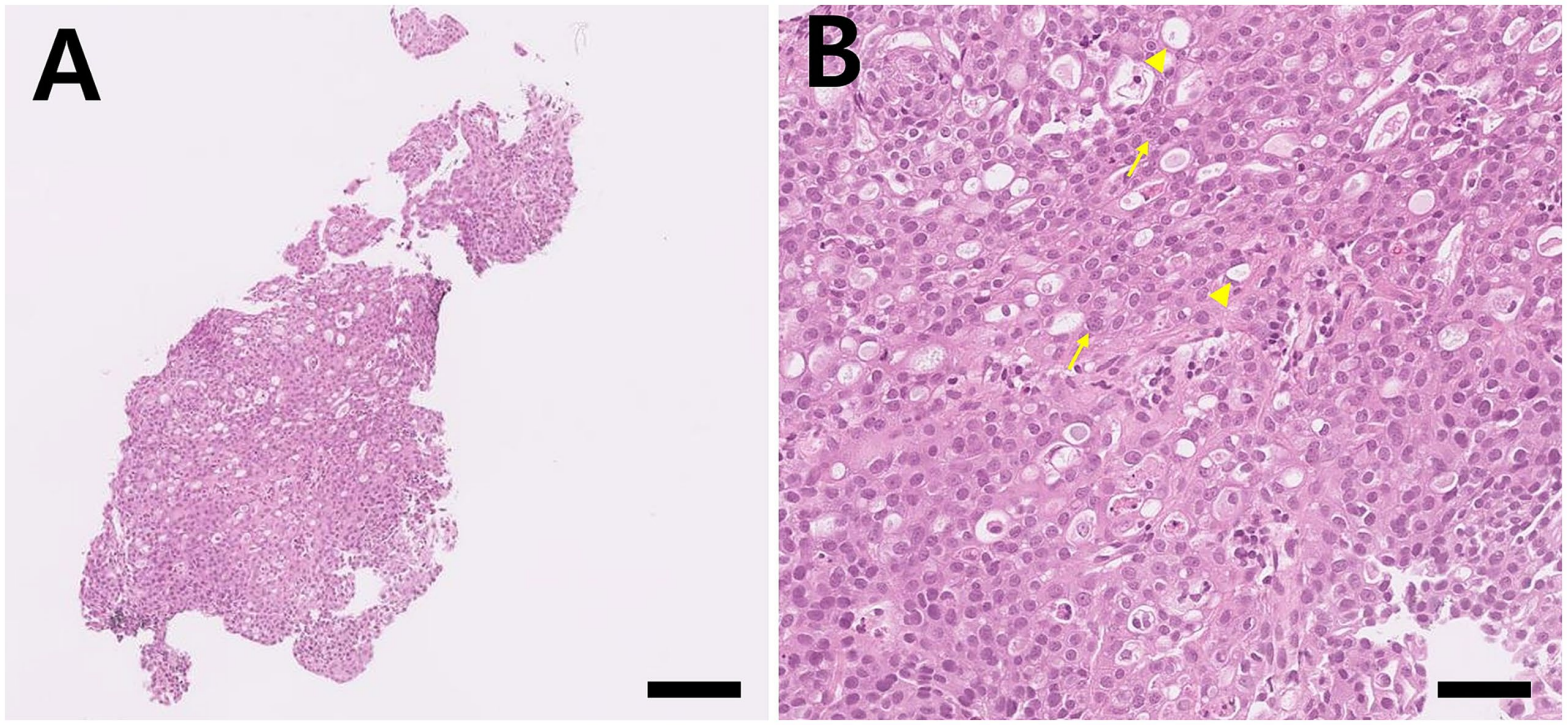
Histopathologic examination of the cystoscopic biopsy specimen in Case 1. **(A)** The submitted tissue consists of urothelium with hyperplastic and dysplastic changes (H&E stain, ×100; scale bar = 400 μm). **(B)** Urothelial cells show nuclei with multiple small nucleoli (yellow arrow) and prominent cytoplasmic vacuolation (arrowhead) (H&E stain, ×400; scale bar = 40 μm).

Under general anesthesia, biopsy of the mass was performed through cystoscopy (Karl-Storz Biopsy Forceps, Karl Storz SE & Co. KG, Tuttlingen, Germany) and the sample was fixed in 10% neutral-buffered formalin, routinely processed, and stained with hematoxylin and eosin (H&E). Histologically, the biopsy consisted entirely of hyperplastic and dysplastic urothelium, with numerous foci of atypical urothelial cells (Figure 2A). Tissue orientation and overall architecture were limited due to the small size of the sample. Urothelial cells were polyhedral and had moderate to occasionally abundant amounts of amphophilic, lightly vacuolated cytoplasm. Individual cells contained large vacuoles filled with basophilic mucinous material. Nuclei were round to oval and variably sized with a granular chromatin pattern and a single prominent nucleolus or multiple smaller nucleoli (Figure 2B). The presumptive diagnosis was TCC.

**Figure 3:**
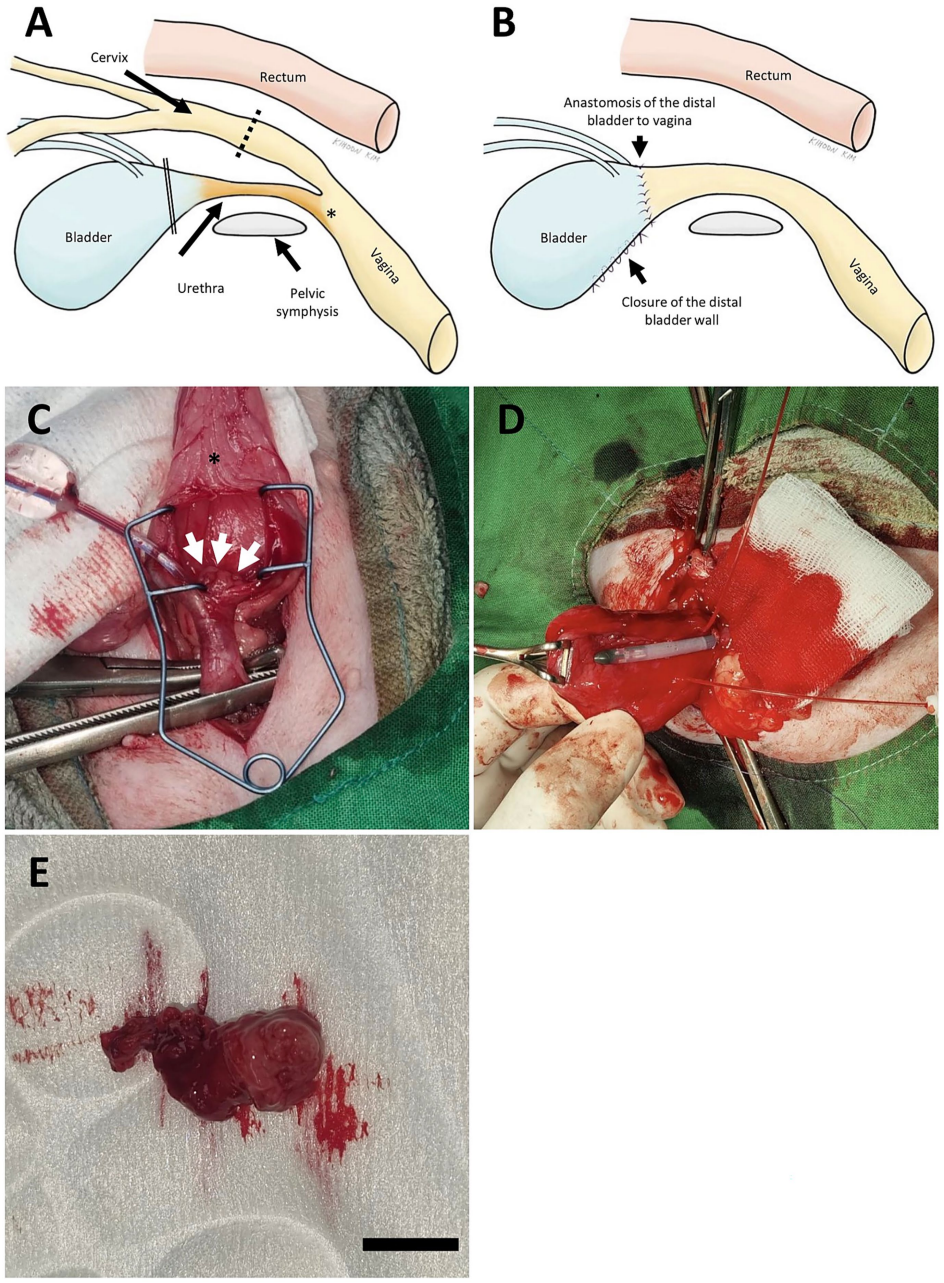
Schematic illustration of vesicovaginal urinary diversion **(A,B)** and intraoperative images in Case 1 **(C,E)** and Case 2 **(D)**. **(A)** Normal anatomical relationship of the female urogenital system. The dashed line indicates the transection site of the vagina and double line indicates the transection site of the distal bladder for the distal bladder to vaginal anastomosis, and the asterisk indicates the site of urethral ligation. **(B)** Surgical reconstruction following total urethrectomy and partial cystectomy. The distal portion of the bladder is anastomosed directly to the vagina to establish urinary diversion, while the remaining distal bladder wall is closed with simple interrupted pattern. This approach preserves the bladder body and ureteral orifices and avoids ureteral reimplantation. **(C)** An oval shaped mass (white arrow) originated from the proximal urethra was identified after incision of the ventral wall of the urinary bladder (asterisk). **(D)** Tomcat catheters placed within the ureter to facilitate visualization and preservation of the ureteral orifices. **(E)** The mass exposed after longitudinal incision of the urethra following total urethrectomy (Scale bar = 1 cm).

With the owner’s consent, total urethrectomy combined with partial cystectomy and vesicovaginal urinary diversion, was elected (Figures 3A,B). Under general anesthesia, ventral midline celiotomy was performed. A ventral cystostomy was performed to expose a mass. On CT imaging, the mass appeared to be adjacent to the left ureteral orifice; however, gross inspection during surgery revealed that the tumor originated from the proximal urethra and protruded cranially. The base of the mass was located approximately 1.5 cm away from the ureteral orifice. (Figure 3C). The caudal end of the urethra at the level of the vulvar vestibule was ligated using 3–0 silk and transected. Tomcat catheter was placed to precisely locate and protect the ureteral orifice in the urinary bladder (Figure 3D, image from Case 2). Subsequently, the bladder neck distal to the ureteral orifice, and along with the entire urethra were removed with efforts to preserve the major blood supply to the bladder. Special care was taken during removal of the distal urinary bladder to preserve the ureteral orifice. The mass was successfully removed with approximately a 1-cm surgical margin of grossly normal tissue. (Figure 3E). Then, the proximal vagina was transected and subsequently anastomosed to the bladder using PDS 4–0 in a simple interrupted suture pattern. No gross residual tumor was evident following resection. After removal of the Tomcat catheter, the remaining cystostomy site was closed using PDS 4–0 in a simple continuous pattern. Routine closure of the abdominal wall and skin was performed. Recovery from the anesthesia was uneventful.

The catheter was removed postoperative day (POD) 4. The patient exhibited persistent urinary incontinence (UI) throughout the follow-up period. The patient was discharged POD 9. Meloxicam (Medicox®, Myungmoon Pharmaceutical, Gyonggi-do, South Korea) was administered orally at 0.05 mg/kg once daily for 2 months, and thereafter the medication was continued at the referring veterinary hospital. Chemotherapy was recommended but declined by the owner due to financial reasons. Follow-up examinations were performed at 2 weeks, 1 month, 2 months, 3 months, and 1 year postoperatively using owner-reported history and ultrasonographic follow-up. Postoperatively, no obstruction of the ureteral orifices was observed. Despite the owner’s initial acceptance, the condition persisted, ultimately requiring the use of diapers for daily management. There was no recurrence of TCC 1 year after the surgery.

### 2.2 Case 2

A 12-year-old, spayed female, weighing 5.1 kg, Shih-Tzu was presented to Nowon N Animal Medical Center for routine check-up. During history taking, the owner reported that the patient had experienced hematuria over the past several months. Blood examinations including complete blood count and electrolytes were within normal limits. However, serum chemical analysis revealed an increased alkaline phosphatase (ALKP, 2149 U/L, reference range: 47–254) level. On abdominal radiologic examination, the bladder neck and proximal urethra were difficult to evaluate due to superimposition by the pelvis. No other remarkable findings were identified. On subsequent ultrasonographic examination, irregular thickening of the entire bladder wall was observed, with a small amount of gravity-dependent, highly echogenic material. Within the urethral lumen, a calcified, proliferative structure measuring approximately 33 × 7 × 6 mm was identified, extending up to the level of the bladder neck.

Under general anesthesia, computed tomographic examination revealed diffusely thickened urethral wall. Pre-contrast images demonstrated high-attenuated calcifications along the distal bladder neck and proximal urethral wall. Post-contrast images revealed a homogeneously enhanced, proliferative mass involving the entire urethra extending to the bladder neck, measuring approximately 30 × 6 × 9 mm. The mass did not involve either ureteral orifice. Mild enlargement of the right sublumbar lymph node was identified compared to the left. No evidence of other abdominal organ involvement was identified. Cystoscopic biopsy of the mass was attempted, but limited access necessitated performing the biopsy through a laparotomy. Therefore, diagnostic incisional biopsies were obtained via an exploratory abdominal approach. The specimen was fixed in 10% neutral-buffered formalin, routinely processed, and evaluated on H&E-stained sections.

Histologically, the lesion was poorly demarcated and consisted of an infiltrative epithelial proliferation extending into the submucosa. Muscularis propria was not included in the specimen, precluding assessment of deeper invasion. The proliferating cells formed multiple layers resembling transitional epithelial cells and exhibited structures similar to tubules. Some cells contained large cytoplasmic vacuoles, and nuclei were displaced peripherally, consistent with signet ring cell morphology. Most cells demonstrated a high nuclear-to-cytoplasmic ratio, with moderate to severe nuclear and nucleolar atypia. Multinucleated cells with two or more nuclei were also observed. Mitotic figures were readily identified, with a mitotic index of approximately 4 per 10 high-power fields. Surgical margin assessment was not performed due to preoperative incisional biopsy sampling. Final diagnosis was TCC.

Surgery was performed 2 weeks after the surgical biopsy using the same technique as described in Case 1. The patient recovered uneventfully from anesthesia. Postoperatively, the 8-Fr catheter was removed 6 days after surgery. The patient was discharged 7 days after surgery. Firocoxib (Previcox®, Merial, Duluth, GA, USA) was administered orally at 2 mg/kg twice daily at discharge and has been continued since then. Chemotherapy was performed a total of six times by administering mitoxantrone (5 mg/m^2^) intravenously at 3-week intervals. No evidence of recurrent TCC was observed one and a half years after the surgery. The patient’s clinical status was assessed monthly after surgery based on owner-reported history and ultrasonographic examinations. During the evaluation period, the patient consistently exhibited UI, which required management using diapers. Ultrasonographic findings were consistent with cystitis and vaginitis throughout the follow-up period; the anastomosis between the bladder and vagina was clearly identified 1 year after surgery (Figure 1C).

## 3 Discussion

Generally, the definitive diagnosis of TCC is made by histopathologic evaluation of tissues (13). In Case 1, the specimen exhibited dysplastic urothelium characterized by architectural disarray and nuclear atypia. In Case 2, the specimen demonstrated an infiltrative epithelial proliferation extending into the submucosa accompanied by marked nuclear atypia and mitotic figures. However, it should be noted that this study is limited the lack of postoperative histopathology and surgical margin assessment, despite tissue samples being obtained via cystoscopy and laparotomy. Furthermore, as cystoscopic biopsy samples are often limited in size, they may not always permit comprehensive histopathological grading. While diagnostic uncertainty or the possibility of misclassification cannot be entirely ruled out, an alternative diagnosis was considered highly unlikely when clinical, imaging, and histopathological findings were collectively evaluated.

Although chemotherapy has been widely accepted as the mainstay of treatment for TCC, various surgical techniques have also been reported (1). Some studies have focused on total cystectomy for the following reasons: to eliminate the source of pain, reduce the risk of urethral obstruction, and prevent or limit future metastasis by removing the primary tumor (9, 14). However, total cystectomy inevitably necessitates ureteral reimplantation, thereby increasing the risk of ureteral obstruction or dehiscence at the anastomosis site (9). Moreover, UI after total or partial cystectomy is a major complication that may discourage radical surgical resection (1, 11). UI results from disruption of urethral length, wall elasticity, muscle tone, or their neural control, with autonomic fibers being particularly vulnerable during periurethral dissection (15, 16). Moreover, the intrinsic antibacterial properties of the urethral mucosa are compromised during urethral resection (10). Palliative modalities such as permanent cystostomy catheter placement, insertion of a self-expanding urethral stent, or cystoscopic laser debulking to restore urinary outflow may be considered for urethral obstruction, notwithstanding their association with substantial morbidity (1, 4, 17–20). In the present cases, surgical approach of total urethrectomy combined with partial cystectomy was selected for the surgical management of the urethral TCC. Total urethrectomy may have provided sufficient surgical margins, although a formal evaluation was omitted in this study. Furthermore, in contrast to total cystectomy, partial cystectomy does not require manipulation of the ureteral orifices, thereby reducing the potential risk of common complications associated with subsequent ureteral reimplantation.

With respect to urinary diversion for the treatment of TCCs, several techniques following total cystectomy have been described in veterinary medicine (4, 11, 16, 21). In one report, ureterocolonic anastomosis was performed in 10 dogs, in which urinary continence was maintained without the need for an external urine collection device (4). The authors suggested that urinary continence was preserved via anal sphincter control. However, postoperative complications such as gastrointestinal and neurologic signs were observed, which were presumed to be associated with electrolyte and acid–base imbalances secondary to colonic reabsorption of urine (3). As alternative approaches, Boston and Singh reported ureteral anastomosis to the proximal vagina (14), while Fabris et al. and Saeki et al. described urinary diversion by anastomosis of both ureters to the prepuce or vagina (9, 10). Despite these efforts, ureteral diversion might further increase the risk of postoperative complications, particularly ureteral obstruction (8, 9). Previous studies regarding urinary diversion are summarized in Table 1.

**Table 1:** Summary of previously reported surgical techniques and urinary diversion used in dogs with TCC requiring partial or total cystectomy.

Authors	Species	Location	Surgery type	Urinary diversion	Complications
Stone et al. (4)	10 dogs	Bladder, or urethra or both	Total cystectomy with the proximal part of the urethra	Ureter to colon	Hyperammonemic encephalopathy, pyelonephritis No UI
White et al. (21)	1 dogs	Urethra	Partial cystectomy	Urethra to Vagina	Lower urinary tract dysfunction
Saulnier-Troff et al. (1)	1 dog	Bladder	Partial cystectomy with the proximal part of the urethra	Bladder to Urethra	Transient UI
Saeki et al. (9)	2 dogs (male)	Bladder	Total cystectomy	Ureter to Prepuce	Anastomotic dehiscence; Azotemia; Metabolic acidosis
	8 dogs (female)	Bladder	Total cystectomy	Ureter to Vagina	Anastomotic dehiscence; Acute kidney injury; Acute pancreatitis; Ureteral obstruction
Skinner et al. (8)	1 Dog	Bladder	Total cystectomy	Ureter to urethra	UI
Fabris et al. (10)	1 Dog	Bladder	Total cystectomy	Ureter to uterine stump	Not reported

In addition, Saulier-Troff et el. suggested en bloc resection including the trigone and proximal urethra, which carries a substantial risk of bladder necrosis (1). Bladder necrosis occurring 2–10 days after partial cystectomy are presumed to result from injury to the caudal vesical artery (11, 12). Therefore, in this case, efforts were focused on preserving the bladder’s primary vascular supply, the caudal vesical artery arising from the internal pudendal artery. For tumors confined to the urethra, on the other hand, preservation of the urinary bladder has been explored. White et al. reported anastomosis of the proximal urethra to the vagina following excision of a urethral TCC in a dog; the patient was euthanized four months after surgery due to local tumor regrowth at the surgical site (21).

In the present report, TCC was successfully managed by a total urethrectomy and partial cystectomy, while preserving both ureteral orifices within the urinary bladder. Intraoperatively, Tomcat catheters were inserted into both ureteral orifices, allowing direct visual identification and protection of the ureteral openings and enabling maximal resection extending to the bladder neck while maintaining ureteral integrity. In cases where the tumor involves not only the bladder trigone but also extends into the distal urethra, the proximal urethrectomy described by Saulinier-Troff et al. (1) may be insufficient. The present cases describe a more aggressive resection including the entire urethra, followed by vesicovaginal urinary diversion. To the authors’ knowledge, this report is the first to describe successful surgical management of TCC involving the bladder neck and urethra, achieved without ureteral reimplantation, by diverting urinary flow from the bladder to the vagina.

Given that the resection included both the entire urethra and the distal portion of the bladder, no evidence of local tumor recurrence has been observed in either case for one year (Case 1) and one and a half years (Case 2) after surgery, respectively. Despite the lack of surgical margin evaluation, the favorable clinical outcome in Case 1, achieved without adjuvant chemotherapy, suggests that the surgical procedure alone provided effective local tumor control. This highlights the potential therapeutic value of total urethrectomy combined with partial cystectomy. Even if the resection was incomplete, the surgical procedure achieved substantial cytoreduction, which likely played a critical role in long-term local disease control. While non-steroidal anti-inflammatory drugs administration for potential antitumor effects in both cases and adjuvant chemotherapy in Case 2 may have contributed to the management of microscopic residual disease (22, 23), the favorable clinical outcome appears to primarily reflect the efficacy of the surgical intervention; according to the previous literature, the mean survival time (MST) of the patients underwent incomplete tumor resection was 75 ~ 120 days (24). Moreover, MST of the patient underwent chemotherapy alone (cisplatin) and in combination with non-steroidal anti-inflammatory drugs (cisplatin and firocoxib) were 338 days, and 179 days, respectively (23). These findings suggest that early surgical management of localized TCC using partial cystectomy combined with complete urethral resection, performed prior to metastatic progression, may provide meaningful clinical benefit in affected dogs. However, additional cases are required to better standardize the association between this surgical approach and long-term clinical outcomes.

UI was consistently observed postoperatively in the present cases and was considered an inevitable consequence of the surgical procedure. In addition to the UI described in the present cases, information on other potential complications remains limited, as vesicovaginal urinary diversion has not been previously described. However, based on previously reported cases in which the vagina served as the terminal conduit for urinary outflow, several potential complications including obstruction and leakage at the anastomosis site may be expected (9). Saeki et al. described surgical site dehiscence, which they attributed to procedural immaturity during the early phase of their surgical experience. Therefore, they recommended temporary catheterization to support the reconstructed urinary tract, minimize tension at the anastomotic site (9), and protect anastomosis from urine exposure during early healing phase (25). Similarly, in cases of vesicovaginal urinary diversion, the use of a urethral catheter for a limited postoperative period should also be considered to reduce the risk of dehiscence.

This study has several limitations, including the absence of regional lymph node excision and postoperative histopathology including margin evaluation, which might have limited accurate staging and prognostic assessment. Further case accumulation is necessary to better delineate and standardize the association between total urethrectomy and partial cystectomy combined with vesicovaginal diversion and long-term clinical outcomes.

## 4 Conclusion

Total urethrectomy combined with partial cystectomy and vesicovaginal urinary diversion achieved effective local tumor control in two dogs with urethral TCC. Despite limited surgical margin assessment, both cases showed prolonged recurrence-free survival, suggesting that early removal of the tumor might provide clinical benefit in those patients with urethral TCC. Persistent UI was observed postoperatively and was considered an inevitable outcome of the procedure but remained acceptable to the owners. Although long-term complications of this surgical approach require further evaluation, this technique might be a feasible surgical option in female dogs with TCC localized to the urethra.

## Data Availability

The original contributions presented in the study are included in the article/supplementary material, further inquiries can be directed to the corresponding author.
